# Death Acceptance and Preparation: Their Roles in Life Satisfaction Among Older Adults Perceiving Health Declines

**DOI:** 10.1093/geroni/igaf122.344

**Published:** 2025-12-31

**Authors:** Yaeji Kim-Knauss, Frieder Lang

**Affiliations:** Friedrich-Alexander-Universität Erlangen-Nürnberg, Nuremberg, Bayern, Germany; Friedrich-Alexander-Universität Erlangen-Nürnberg, Nuremberg, Bayern, Germany

## Abstract

Confronting mortality is an inevitable aspect of aging. This study distinguishes two strategies older adults may employ to cope with life’s finitude: death preparation (an assimilative, instrumental approach) and death acceptance (an accommodative approach). We examined whether these strategies differently predict life satisfaction, particularly when individuals perceive declining health. Data were drawn from the longitudinal Ageing as Future online study in Germany (2012–2023), collected at two-year intervals. A total of 664 older adults (aged 60–89 at baseline) contributed 1,592 observations in total. Using linear mixed modeling, we tested how engagement in death acceptance and death preparation (i.e., having a living will and/or having designated power of attorney) predicted life satisfaction at both between- and within-individual levels, depending on subjective health. We also explored whether the interplay of these approaches provides additional protective effects under deteriorating health conditions. Results showed that, although the presence of death preparation alone was significantly associated with higher life satisfaction, it was not moderated by subjective health. In contrast, at both between- and within-individual levels, individuals who accepted death showed greater life satisfaction, especially when health perceptions were poorer. Notably, this protective effect of death acceptance was most pronounced among those without death preparation in place. Our findings indicate that, under conditions of declining health, death acceptance, conceptualized as an accommodative coping strategy, seems particularly adaptive. Therefore, fostering psychological preparedness and personal meaning in one’s remaining time may be beneficial as older adults face increasing aging-related challenges.

